# Toxicity of biogenic zinc oxide nanoparticles to soil organic matter cycling and their interaction with rice-straw derived biochar

**DOI:** 10.1038/s41598-021-88016-x

**Published:** 2021-04-19

**Authors:** Shemawar, Abid Mahmood, Sabir Hussain, Faisal Mahmood, Muhammad Iqbal, Muhammad Shahid, Muhammad Ibrahim, Muhammad Arif Ali, Tanvir Shahzad

**Affiliations:** 1 Department of Environmental Sciences and Engineering, Government College University Faisalabad, Allama Iqbal Road, Faisalabad, 38000 Pakistan; 2 Department of Bioinformatics and Biotechnology, Government College University Faisalabad, Allama Iqbal Road, Faisalabad, 38000 Pakistan; 3 Department of Soil Science, Bahauddin Zakariya University, Multan, Pakistan

**Keywords:** Environmental sciences, Biogeochemistry, Carbon cycle

## Abstract

Given the rapidly increasing use of metal oxide nanoparticles in agriculture as well as their inadvertent addition through sewage sludge application to soils, it is imperative to assess their possible toxic effects on soil functions that are vital for healthy crop production. In this regard, we designed a lab study to investigate the potential toxicity of one of the most produced nanoparticles, i.e. zinc oxide nanoparticles (nZnO), in a calcareous soil. Microcosms of 80 g of dry-equivalent fresh soils were incubated in mason jars for 64 days, after adding 100 or 1000 mg of biogenically produced nZnO kg^−1^ soil. Moreover, we also added rice-straw derived biochar at 1 or 5% (w: w basis) hypothesizing that the biochar would alleviate nZnO-induced toxicity given that it has been shown to adsorb and detoxify heavy metals in soils. We found that the nZnO decreased microbial biomass carbon by 27.0 to 33.5% in 100 mg nZnO kg^−1^ soil and by 39.0 to 43.3% in 1000 mg nZnO kg^−1^ soil treatments across biochar treatments in the short term i.e. 24 days after incubation. However, this decrease disappeared after 64 days of incubation and the microbial biomass in nZnO amended soils were similar to that in control soils. This shows that the toxicity of nZnO in the studied soil was ephemeral and transient which was overcome by the soil itself in a couple of months. This is also supported by the fact that the nZnO induced higher cumulative C mineralization (i.e. soil respiration) at both rates of addition. The treatment 100 mg nZnO kg^−1^ soil induced 166 to 207%, while 1000 mg nZnO kg^−1^ soil induced 136 to 171% higher cumulative C mineralization across biochar treatments by the end of the experiment. However, contrary to our hypothesis increasing the nZnO addition from 100 to 1000 mg nZnO kg^−1^ soil did not cause additional decrease in microbial biomass nor induced higher C mineralization. Moreover, the biochar did not alleviate even the ephemeral toxicity that was observed after 24d of incubation. Based on overall results, we conclude that the studied soil can function without impairment even at 1000 mg kg^−1^ concentration of nZnO in it.

## Introduction

During recent decades, engineered nanoparticles (NPs) have gained widespread popularity for their use in agriculture and allied sectors due to their specific characteristics^1,2^. The Food and Agriculture Organization & the World Bank are also promoting their use in agriculture^3^. This may lead to an exponential increase in use of NPs in agriculture sector. Consequently, their load in agricultural soils is expected to rise. Currently, inadvertent application of NPs in sewage sludge is the main route of NPs’ entry to agricultural soils^4^. Increasing use of NPs in agricultural inputs like fertilizers and pesticides would become another source of NPs load in soils in future^5,6^. The input of NPs in agricultural soils can affect soil fauna and flora and thereby the associated agroecosystem services^1, 7–9^.

Metal oxide nanoparticles are a major class of NPs that are used in a wide range of products. In a recent synthesis of literature, metal oxide NPs have been reported as one of the most toxic NPs to soil biota^8^. This may cause short to long term loss of soil health and fertility since soil biota are key to these services^10–12^. Numerous studies have been carried out to assess the potential toxic effects of metal oxide NPs on soil invertebrates & microorganisms^8,10^. However, studies exploring the effect of metal oxide NPs on the processes that determine soil health & fertility i.e. soil organic matter decomposition, nutrient cycling, and enzymatic activity are a few.

Among metal oxide NPs, zinc oxide (ZnO) nanoparticles have one of the highest production volumes^13,14^. They are mainly used in different products like cosmetics, medicines, food and solar cells. Owing to their antimicrobial & antigenic properties, they are widely used for such services like veterinary sciences, food storage, and killing microbes to enhance shelf life of food products^8,15^. Therefore, they may be toxic when released in the environment. Given their widespread use and potential to contaminate soils, it is imperative that their potentially toxic effect on soil processes is determined across different soils.

Biochar, a carbonaceous material made by pyrolyzing organic waste materials, is being promoted as a soil conditioner to enhance soil fertility, water holding capacity, nutrient retention, and soil C storage^16–18^. In addition, biochar has also been shown to decrease the toxicity of heavy metals in soil^19^. Since adsorption capacity of biochar is much higher than its precursors, the leachability and bioavailability of heavy metals can be reduced because biochar has large surface area to adsorb heavy metals and organic pollutants^20^. It can, therefore, be imagined that the biochar materials would also be capable of adsorbing metals derived from metal-oxide nanoparticles thereby reducing their toxicity^21^.

Although the potential toxicity of NPs in soil processes has been studied to some extent recently, such kind of studies are rare in calcareous soils. Moreover, the use of biochar to reduce the potential toxicity of NPs has rarely been explored. To fill this gap, we designed a study to explore the effect of *n*ZnO (100 & 1000 mg *n*ZnO kg^−1^ soil) on soil processes related to soil health in a calcareous soil in a lab incubation. We explored following questions: i) how will *n*ZnO affect soil processes related to C and N cycling in an organic-matter poor, calcareous soil and, ii) if the *n*ZnO is toxic to soil processes, will the addition of biochar alleviate that toxicity? We hypothesized that the *n*ZnO NPs would negatively affect the soil processes and the increasing nZnO addition would further toxify the soil processes. We also hypothesized that the rice-straw derived biochar would decrease the toxic effects of *n*ZnO on soils processes.

## Results

### Characteristics of the nZnO

The Field Emission Scanning Electron Microscope (FESEM) analysis showed that the nZnO synthesized and used in this study were having granular shape agglomerated particles in the size range of 90–110 nm (Table 1, Fig. S1). The dynamic light scanning technique revealed that the nZnO carried a negative zeta potential of -27.41 mV (Table 1). The FT-IR analyses revealed the OH stretching of intramolecular hydrogen bond, C = C bond stretching and C–C stretching of Alkanes at peaks obtained at 3740 cm^−1^, 1644 cm^−1^, 1429 cm^−1^ and 1013 cm^−1^^22,23^. Importantly, the stretching vibrations of ZnO bond were indicated in the peak obtained at 522 cm^−1^^24^(Figure S2). The XRD pattern showed characteristic peaks of nZnO at 2θ = 32.3˚, 35.2˚, 37˚, 48.3˚, 57.4˚, 63.6˚, 66.9˚, 68.9˚, 70˚, 73.3˚, 77.6˚ corresponding to (100), (002), (101), (102), (110), (220), (103), (112), (201), (004) and (311) planes (Fig. S3), respectively, and the data are matched well with those reports in literature and the Joint Committee on powder diffraction standards (JCPDS) file No. 04-0783^25^.

**Table 1:** Pre-incubation physiochemical characteristics of soil, rice-straw derived biochar (BC) and zinc oxide nanoparticles (nZnO).

Parameter	Soil	Rice-straw derived Biochar (BC)	Zinc oxide nanoparticles (nZnO)
Sand (%)	50.33 ± 1.76		
Silt (%)	22.67 ± 2.33		
Clay (%)	26.67 ± 0.33		
Textural Class	Sandy clay loam		
pH	8.25 ± 0.04	8.97 ± 0.01	
Electrical conductivity (d S m^−1^)	0.37	1.34	
Organic C (g kg^−1^ dry matter)	6.65 ± 0.53	281.9 ± 11.2	
Total N (g kg^−1^ soil)	0.57 ± 0.05	7.41 ± 0.97	
C/N	11.7	38.04	
Size (nm)			90–110
Shape			Granular agglomerated particles
Zeta potential (mV)			– 27.41

### Soil pH

**Figure 1:**
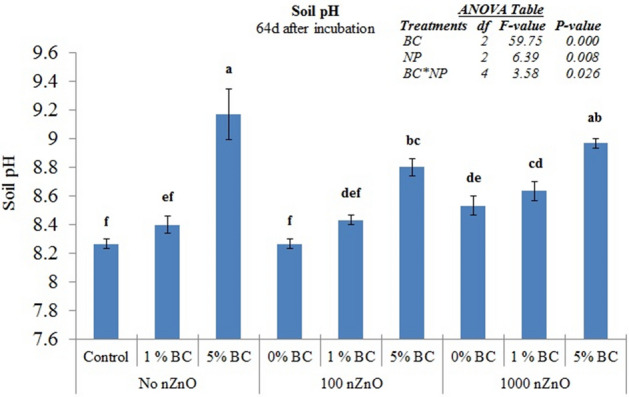
Soil pH as affected by zinc oxide (nZnO) and rice-straw derived biochar after 64d of incubation. Different small letters on top of bars represent post-hoc HSD difference at 95% confidence intervals. Error bars represent standard errors of means (n = 3).

Application of zinc oxide nanoparticles (nZnO) as well as the rice-straw derived biochar (BC) significantly increased the soil pH (Fig. 1, *P* < 0.05). Addition of BC at 5% induced the largest increase in soil pH, i.e. by 0.53 to 0.9, irrespective of the nZnO addition. The nZnO application caused an increase in soil pH only at 1000 mg kg^−1^ soil application. The BC and nZnO had significant interactive effect on soil pH.

### Carbon mineralization

**Figure 2:**
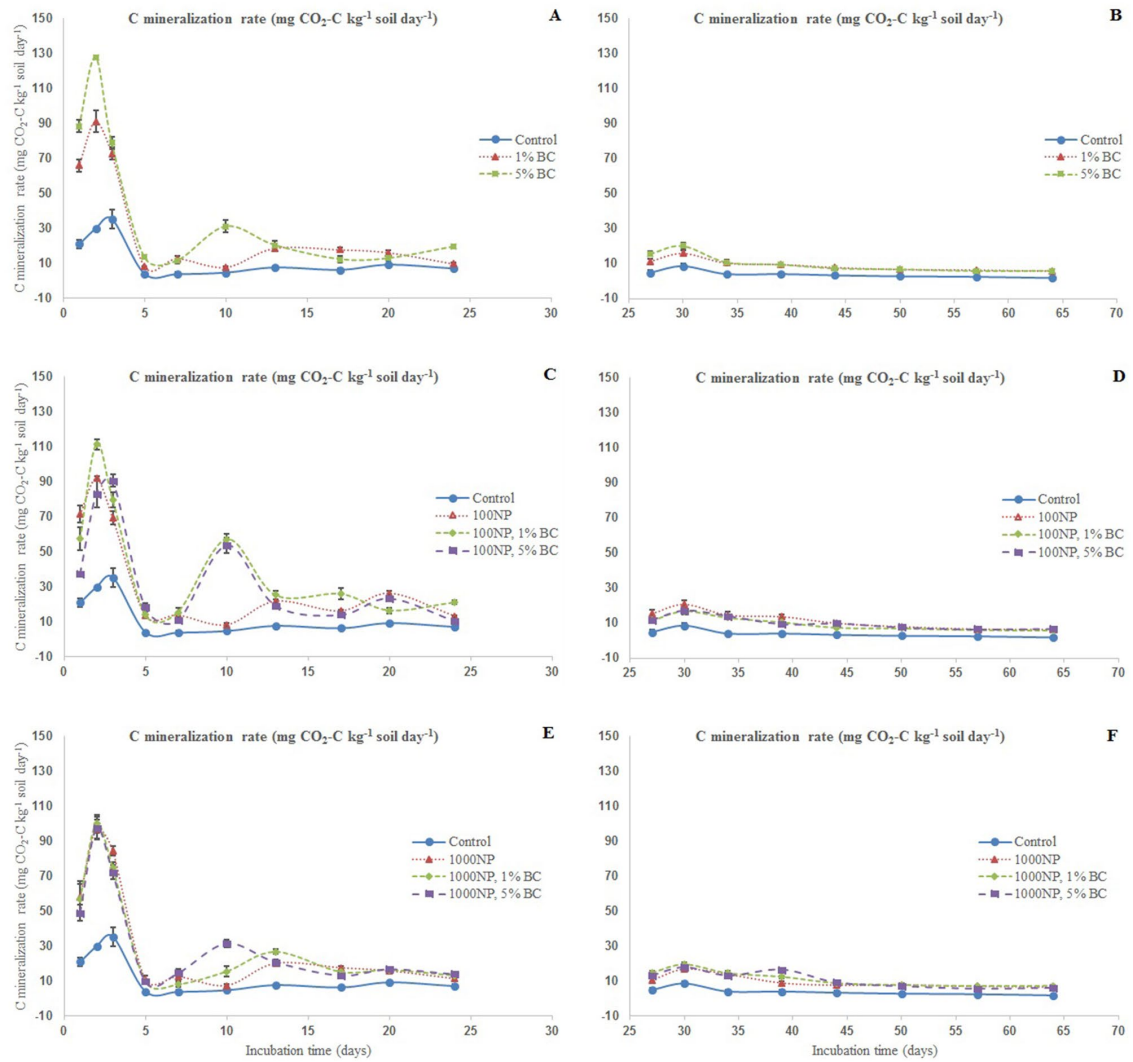
Carbon mineralization rate (mg CO_2_-C kg^−1^ soil day^−1^) as affected by zinc oxide (nZnO) and rice-straw derived biochar after 24d (panels **A**, **C**, **E**) & 64d (**B**, **D**, **F**) of incubation. C mineralization in different BC, nZnO and BC + nZnO treatments for a given date have been plotted in different panels (e.g. panels **A**, **C**, **E**) for the purpose of simplification.

Carbon mineralization significantly changed in response to both treatments i.e. nZnO & BC addition as well as with the time of incubation (Fig. 2, *P* < 0.05). The lower addition of nZnO (i.e. 100 mg nZnO kg^−1^ soil) induced higher C mineralization (mg CO_2_-C kg^−1^ soil day^−1^) than the higher nZnO addition (i.e. 1000 mg nZnO kg^−1^ soil). However, both the biochar addition levels induced similar increase in C mineralization overall. There were two distinct phases of C mineralization i.e. first phase over 0–24 days of incubation (Fig. 2A, C, E) & the second phase spread over 25–64 days of incubation (Fig. 2B, D, F). Briefly, the treatments-induced increase was very high in the initial phase. For some measures, both nZnO & BC had synergistic effect to increase the C mineralization e.g. adding biochar with 100 mg nZnO kg^−1^ soil significantly increased C mineralization than their individual effects. The increase in C mineralization tapered off from 20th day of incubation onwards in a way that the treatments did not differ with the control soil in terms of C mineralization from 45th day of incubation.

**Figure 3:**
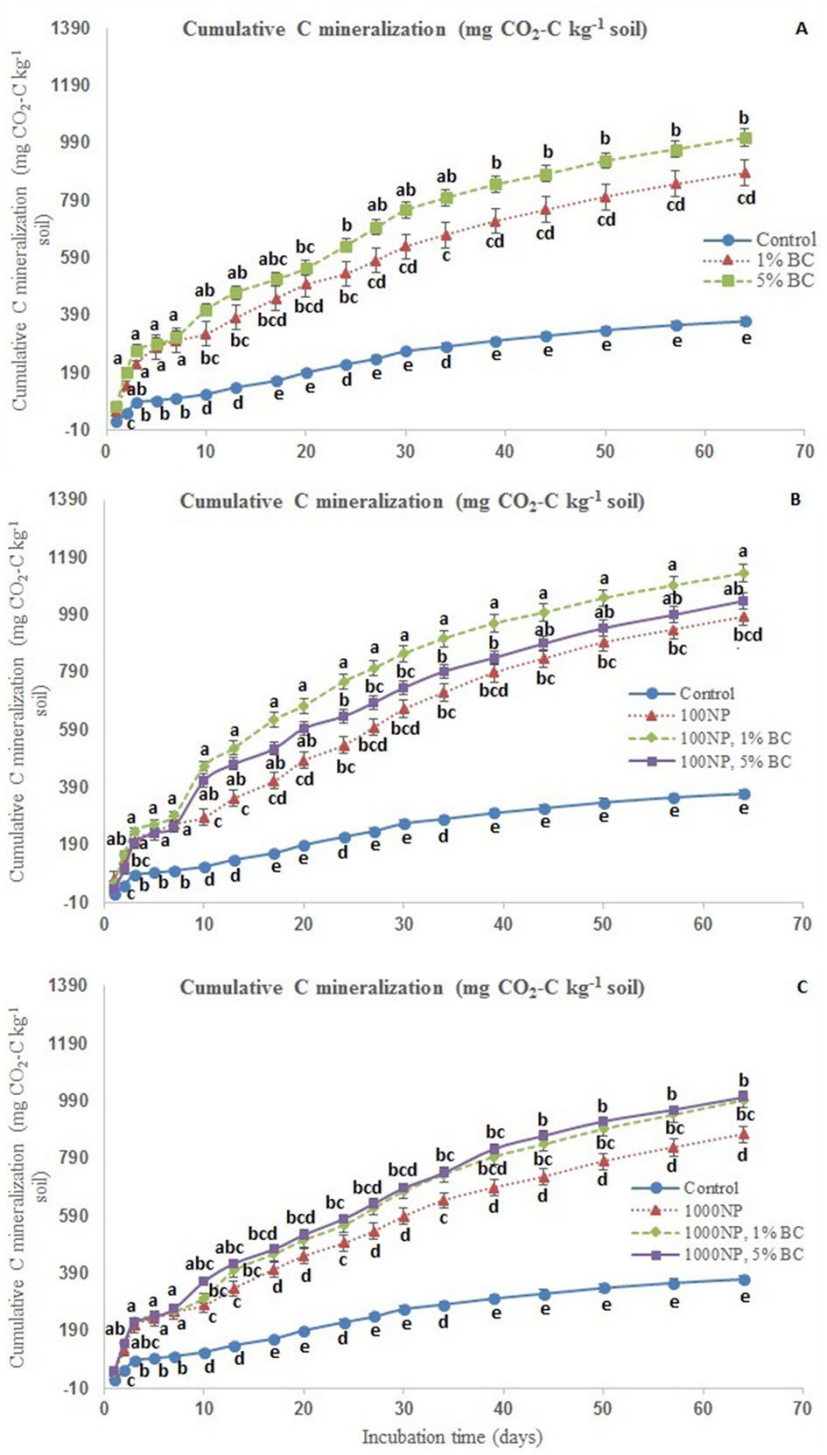
Cumulative carbon mineralization (mg CO_2_-C kg^−1^ soil) as affected by zinc oxide (nZnO) and rice-straw derived biochar over 64 days of incubation. Cumulative C mineralization in different BC, nZnO and BC + nZnO treatments for a given date have been plotted in different panels (e.g. panels **A**, **C**, **E**) for the purpose of simplification. Different small letters on treatment points across three panels represent post-hoc HSD difference at 95% confidence interval.

As a corollary to C mineralization rates, the cumulative C mineralization was significantly higher for both amendments when compared to control soils (Fig. 3, *P* < 0.05). The treatment 100 mg nZnO kg^−1^ soil induced 166 to 207% while 1000 mg nZnO kg^−1^ soil induced 136 to 171% higher cumulative C mineralization across biochar treatments by the end of the experiment. The lower nZnO addition induced higher cumulative CO_2_-C release across all the treatments. However, both the BC treatments i.e. 1 & 5% addition did not differ in terms of final cumulative CO_2_-C release. The interactive effect of nZnO & BC was the most prominent when 1% BC was added to 100NP treatment (100NP 1% BC) such that this treatment showed highest cumulative C mineralization from 10th day of incubation till the end.

### Microbial biomass (MBC), dissolved organic carbon (DOC) & metabolic quotient

Both microbial biomass carbon and dissolved organic carbon were measured twice: first, they were determined after 24 days of incubation whereas second measurement was done at the end of the incubation.

**Figure 4:**
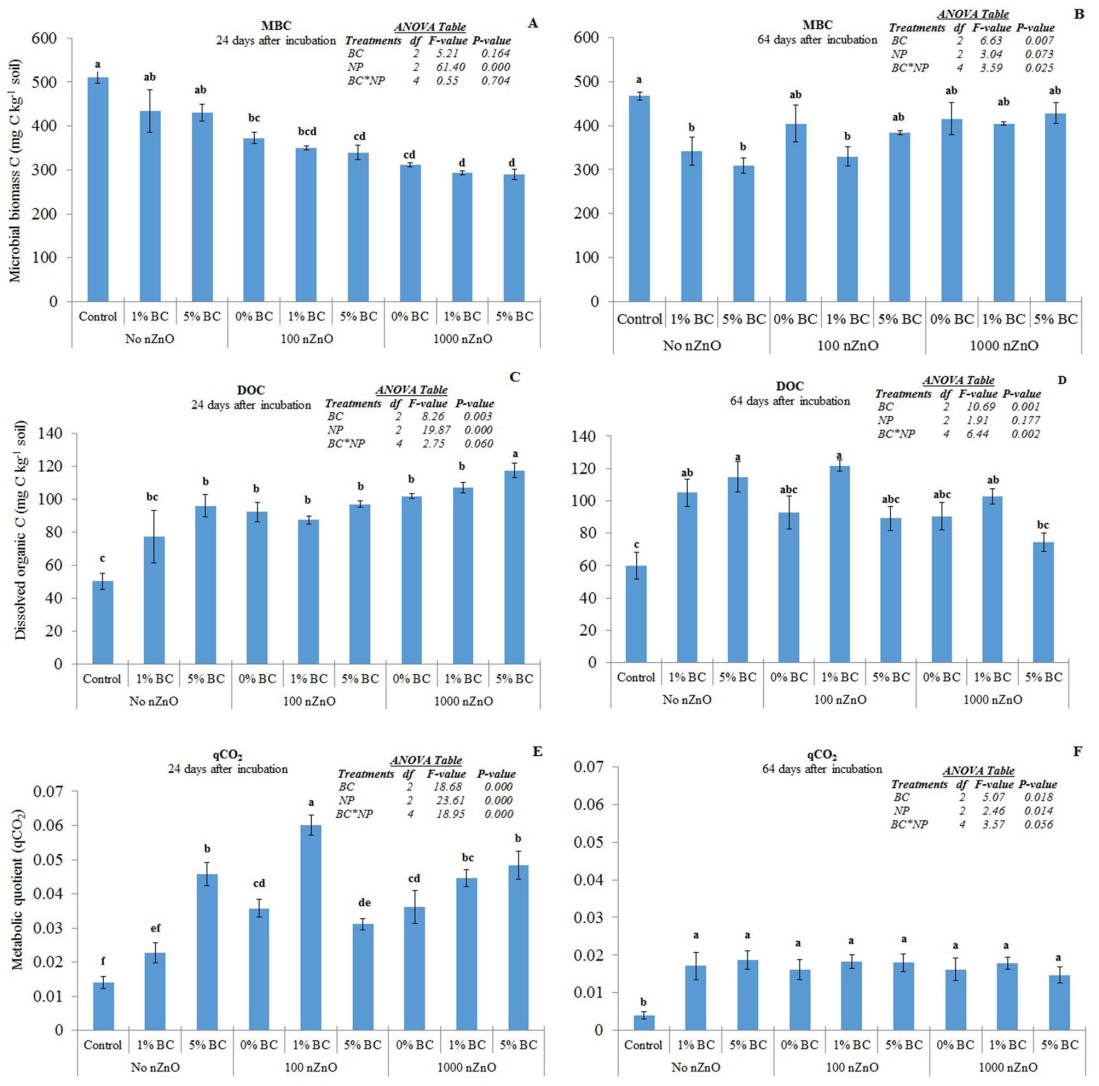
Microbial biomass C, dissolved organic C and metabolic quotient in response to nZnO and BC treatments after 24 (panels **A**, **C**, **E** respectively) and 64 days (panels **B**, **C**, **F** respectively). Different small letters on top of bars represent post-hoc HSD difference at 95% confidence interval. Error bars represent standard errors of means (n = 3).

The MBC was significantly reduced by nZnO at both application rates for the first measure (Fig. 4A, P < 0.05). This decrease was by 27.0 to 33.5% in 100 mg nZnO kg^−1^ soil and by 39.0 to 43.3% in 1000 mg nZnO kg^−1^ soil treatments across biochar treatments. Both levels of nZnO addition induced similar decrease. Biochar did not induce any change in MBC at this stage, nor its interaction with nZnO. At the end of the incubation, no effect of nZnO application on MBC was observed (Fig. 4B). However, BC application at both rates (1 & 5%) significantly reduced the MBC (*P* < 0.05). The BC and nZnO application had a positive interactive effect on MBC such that the MBC increased in 100 nZnO 5% BC, 1000nZnO 1% BC and 1000 nZnO 5% BC treatments when compared to BC (1 & 5%) only treatments.

At 24th day of incubation, the dissolved organic carbon (DOC) was significantly increased by application of zinc oxide nanoparticles as well as biochar (Fig. 4C, *P < 0.05*). The rates of application of both the amendments did not differ in terms of DOC content. Similarly, there was no interactive effect of nZnO and BC. At the end of the incubation, nZnO had no effect on DOC whereas BC only treatments (1 & 5% BC) showed significantly higher DOC content than control (Fig. 4D, *P < 0.05*). The NP and biochar treatments had interactive effect on DOC such that all of their combinations showed DOC content similar to those in control. However, the treatment 100NP 1% BC was an exception where the DOC content was significantly higher than control. The harvest time did not have any effect on DOC.

Metabolic quotient (qCO_2_), CO_2_-C released per unit of microbial biomass, was significantly increased by both nZnO & BC, as well as, their interaction after 24 days of incubation (Fig. 4E). The addition of 1% BC with low nZnO addition caused the highest qCO_2_. After 64 days of incubation too, the qCO_2_ was significantly increased by both amendments (Fig. 4F). However, there was not interactive effect after 64 days of incubation.

### Mineral nitrogen & available phosphorus content

Nanoparticle application significantly changed the mineral NO_3_^−^–N content in soils whereas the biochar application did not affect it (Table 2). Moreover, there was a significant nanoparticle × biochar effect on nitrate content. Unexpectedly, no increase was observed in NO_3_^−^–N content with biochar addition. The addition of nZnO in low amount (i.e. 100 mg nZnO kg^−1^ soil) significantly increased the NO_3_^−^–N content, whereas addition of biochar along with it did not cause any further change. However, increasing the nZnO addition amount to 1000 mg nZnO kg^−1^ soil did not cause any change in the NO_3_^−^–N content with respect to control. Addition of biochar at 5% rate along with 1000 mg nZnO kg^−1^ soil increased the NO_3_^–^N content to those found in 100 mg nZnO kg^−1^ soil treatments, though addition of biochar at 1% did not cause any increase. No treatment effect was observed on NH_4_^+^-N content. Mineral N, calculated by adding NH_4_^+^-N and NO_3_^–^N, was changed by nZnO in the way similar to NO_3_^–^N content.

**Table 2:** Effect of nZnO & BC on NO_3_^–^ N, NH_4_^+^-N and mineral N.

64 Days of incubation			
Treatments	NO_3_^–^ N µg g^−1^ soil	NH_4_^+^-N µg g^−1^ soil	Mineral N
Control	17.5 (3.6)cd*	0.01 (0.0)a	17.5 (3.6)de
1% BC	18.3 (4.0)bc	0.05 (0.01)a	18.4 (4.0)cde
5% BC	19.3 (3.0)abcd	0.06 (0.03)a	19.4 (3.0)bcde
100 nZnO	24.8 (1.0)ab	0.06 (0.02)a	24.9 (1.0)ab
100 nZnO, 1% BC	25.8 (0.6)a	0.04 (0.01)a	25.8 (0.6)a
100 nZnO, 5% BC	24.2 (0.4)ab	0.05 (0.01)a	24.3 (0.4)abc
1000 nZnO	17.2 (0.5)d	0.03 (0.01)a	17.2 (0.5)e
1000 nZnO, 1% BC	14.6 (1.2)d	0.04 (0.0)a	14.6 (1.2)e
1000 nZnO, 5% BC	23.9 (1.7)abc	0.03 (0.01)a	23.9 (1.7)abcd

Different letters within a column indicate significant difference between means at 95% confidence interval. The brackets contain standard errors of means (n = 3).

**Figure 5:**
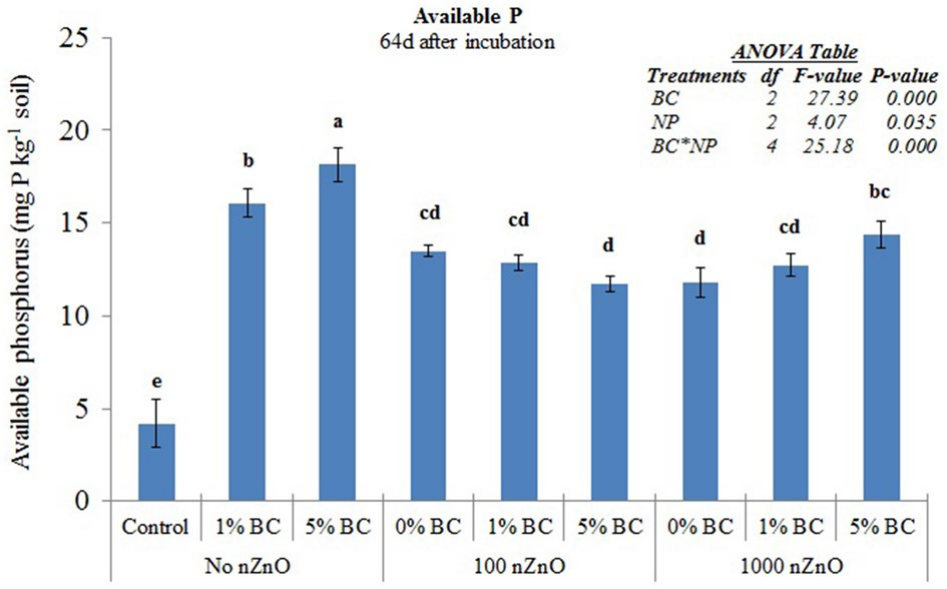
Available phosphorus in soils in response to nZnO and BC treatments 64d after incubation. Different small letters on top of bars represent post-hoc HSD difference at 95% confidence interval. Error bars represent standard errors of means (n = 3).

Both the treatments, ZnO nanoparticles as well as biochar, induced significant increase in available P (Fig. 5). The highest increase was observed in biochar only treatments. Although, NP treatments also induced significant increase in P availability, their combination with BC did not show any additional increase, rather it decreased the available P when compared to biochar only treatments.

### Bacterial colony forming units

**Figure 6:**
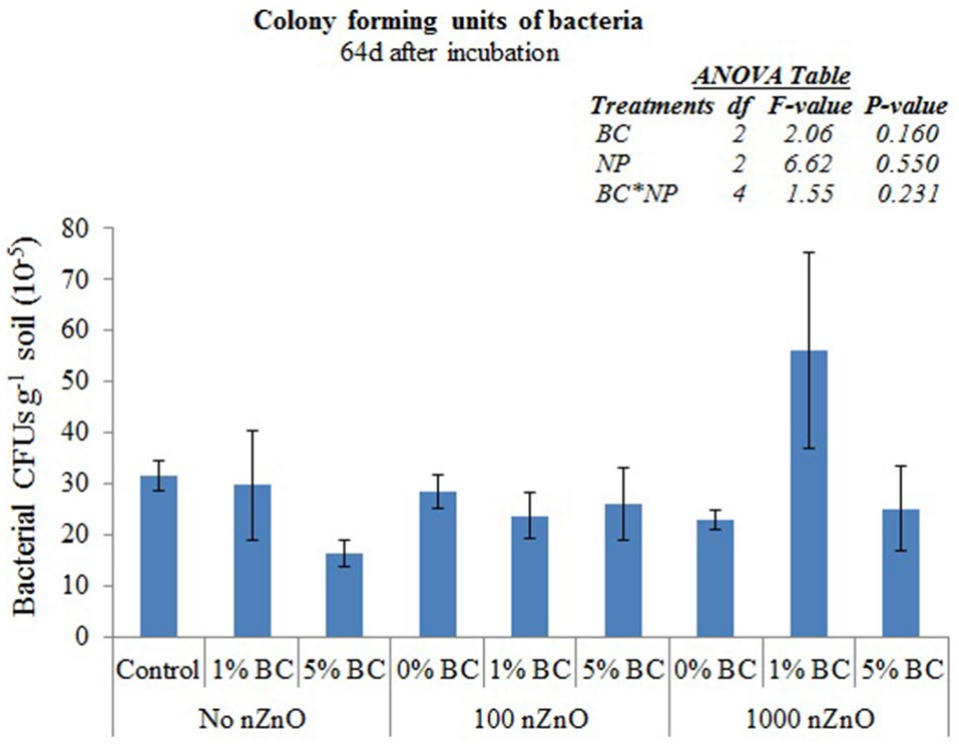
Colony forming units (CFUs) of bacteria found in soils in response to nZnO and BC treatments 64d after incubation. Error bars represent standard errors of means (n = 3).

None of the two treatments affected bacterial colony forming units (CFU) (Fig. 6, *P* > 0.05).

## Discussion

ZnO nanoparticles (nZnO) significantly reduced the microbial biomass carbon (MBC) in the short term, i.e. when measured on 24th day of incubation (Fig. 4A). This result partially proves our first hypothesis i.e. the nZnO induced toxicity in our soil. Reduced microbial biomass was observed in a grassland soil 15 & 60 days after application of nZnO^26^. Moreover, they found an exponential decrease in MBC with increasing concentration of nZnO (i.e. 50, 100 & 500 mg kg^−1^ soil). However, in our study, increasing the nZnO concentration from 100 to 1000 mg kg^−1^ soil did not cause further decrease in MBC. This is intriguing though not unexpected given the overall ephemeral toxicity of the nZnO observed in this study. In a previous study, toxicity of nZnO doses vis-à-vis microbial biomass was dependent on sensitivity of the method used to estimate the microbial biomass. The DNA extraction showed an exponential decrease with increasing dose of nZnO from 50 to 500 mg kg^−1^ soil, whereas substrate-induced respiration, i.e. a method more similar to fumigation-extraction used in this study than the DNA extraction, found no change within nZnO doses after 15 days of exposure^26^. Moreover, the MBC in nZnO amended soils recovered to that found in control after 64 days of incubation in our study. These results indicate that the toxicity of the nZnO used in this study was transient and ephemeral. The unchanged culturable bacteria also support the transient nature of the toxicity induced by nZnO in our soil.

Our second hypothesis was that biochar would mitigate the toxic effects induced by the nZnO. However, this was not the case as the MBC after 24 days of incubation remained lower in nZnO amended soils than the control soils even after addition of 1% or 5% BC (Fig. 4A). The toxicity of nZnO, or any metal oxide nanoparticle for that matter, is related to the extent of its dissolution and subsequent availability of its constituent metal ions to microbes^27,28^. Although we did not measure dissolution of nZnO used in our study nor did we measure the possible microbial uptake of Zn^2+^, this nanomaterial is known for its easy dissolution in soils^29,30^. Moreover, the FESEM and XRD analyses have confirmed the active nano nature of the nZnO particles. Therefore, it can be assumed that the transient toxicity induced by nZnO in our study was due to Zn^2+^ ions released and absorbed by microbes after dissolution of nZnO^26,30,31^. The biochar, being an organic material and known as sorbent for heavy metals, was expected to complex ionic zinc thereby reducing the toxicity. On the contrary, biochar itself decreased MBC by the end of the incubation (Fig. 4B). Addition of biochar to soils usually increases MBC^32^. However, it may not induce any change or reduce the MBC as well^17,33,34^. For instance, rice-straw derived biochar reduced the MBC in another study^34^; a result which corresponds to our study. Volatile organic compounds on the surface of biochars may cause toxicity to microbial biomass^33^.

Both the treatments i.e. nZnO & BC increased the CO_2_-C release from soils (Figs. 2, 3) as well as metabolic quotients, which, over time, tapered off to that coming out of control soils (Figs. 2, 3). In a previous study, nZnO were found to induce no change in soil respiration at 0.1–100 mg kg^−1^ soil addition whereas a reduction at 1000 mg kg^−1^ soil addition was observed^35^. However, activity of dehydrogenase, cellobiohydrolase, xylosidase & glucosidase enzymes, which are linked with CO_2_-C release, were found to increase after exposure to nZnO^28,29,36^. The increase in CO_2_-C release in response to heavy metal ions (i.e. Zn^2+^ in our study) can be the result of two processes: turnover of microbial biomass & stress induced increases in metabolic quotients^31,37^. Until 24th day of incubation in our study, both the processes seem to contribute to increase in respiration in response to nZnO addition given the significant decrease in MBC and very high metabolic quotients. In the second phase, the increase in respiration was nominal thereby resulting in less prominent metabolic quotients. Even if there was no decrease in MBC in the second phase, the microbes are stressed in presence of heavy metal ions, inefficient to use substrates (i.e. biochar in our case) and have to expend more energy for maintenance than assimilation^37^.

Intuitively, increase in dissolved organic carbon (DOC) should increase the microbial biomass^38^. Both nZnO as well as BC induced increase in DOC on both occasions; more prominent during the first phase (Fig. 4C, D). However, this increase in DOC did not translate into increase in MBC. The literature shows that this may not be an anomaly^39^. The increase in DOC in our study may be linked to increase in soil pH by both treatments. In a survey of 33 alkaline soils (i.e. pH > 8) in Australia, DOC was found in a positive relationship with pH suggesting that the increase in pH enhances solubility of DOC and stimulates desorption of DOC from mineral sites^40^.

The response of mineral nitrogen availability to nZnO was enigmatic (Table 2). The 100 mg nZnO kg^−1^ soil treatment induced a significant increase while 1000 mg nZnO kg^−1^ soil did not induce any change in it. This result corresponds to some previous studies. For instance, 10 & 100 mg nZnO kg^−1^ soil significantly increased the *β*-1,4-N-acetylglucosaminidase activity implying an increase in mineral N^29^. However, the nZnO in 10, 100 & 1000 mg nZnO kg^−1^ soil concentrations have been reported to inhibit urease activity thereby implying lower availability of mineral N^28^, whereas no effect of the same have also been reported at same addition rates^35^. However, the decrease in mineral N at 1000 mg nZnO kg^−1^ soil cannot be explained based on our data. The no decrease in soil mineral N in response to biochar only additions is expected given that the microbes may immobilize mineral N present in solution in order to decompose the organic matter i.e. biochar^17,41^. Although these may explain our results to some extent, the effect of metal oxide nanoparticles on nitrogen dynamics in soils warrant detailed investigations. The available phosphorus (P) increased in all the treatments (Fig. 5). It is common for biochar to increase the P availability given that it releases this nutrient on decomposing^32,41^. The decrease in available phosphorus after nZnO addition along with biochar compared to biochar only treatments indicate that there is some kind of restraint put by nZnO on microbes or solubilization process responsible for release of phosphorus in the solution. The composites of metal oxide nanoparticles have been shown to adsorb phosphorus from the aqueous medium, although the same has not been reported for soil so far^42^. Irrespective of the mechanism, this result shows that the nZnO could negate the benefit of nutrient accrual from biochar. As far the available P increase in response to nZnO application, no study have so far reported the effect of nZnO on P availability. However, activities of P cycling enzymes in response to nZnO have been reported. For instance, alkaline phosphatase activity has been found to significantly increase by nZnO application at the concentrations of 10 to 1000 mg nZnO kg^−1^ soil supporting our result^28,29,36^. These authors found that the nZnO indeed inhibited the alkaline phosphatase activity within few hours of application while stimulated it after 30 days of exposure. They suspected that the nZnO might have been complexed by soil organic matter over time. These results combined with our study suggest that the interactions of nanoparticles with nutrient cycling and their mechanistic bases are complicated and need further investigations.

In conclusion, we can say that the biogenically produced zinc oxide nanoparticles showed ephemeral and transient toxicity to microbial biomass in our soil and that this toxicity vanishes after 64 days of incubation. This apparent contradiction of our results with respect to literature is most likely due to the biogenic nature of the nZnO particles used in our study. To our knowledge, this is the first study reporting the toxicity assessment of biogenic metal oxide nanoparticles to soil processes. The agglomerated nature of the biogenic nZnO used in our study could explain their ephemeral toxicity given that the agglomeration renders nanoparticles less toxic by reducing the effective volume ratio and surface area^43,44^. Moreover, contrary to our second hypothesis, the biochar did not alleviate this minor toxicity induced by the nZnO. Overall, we can say that the studied soil can function without impairment even at 1000 mg kg^−1^ concentration of biogenic nZnO in it.

## Methods

### Soil, ZnO nanoparticles and biochar

A surface soil (0–20 cm) was sampled from agronomy farm of University of Agriculture, Faisalabad. The sampled field has been under wheat–maize crop rotation for more than a decade. Five cores were taken from random locations of the selected plot. All the root debris and gravels were removed with hand before sieving the soil through a 2 mm sieve. The soil cores were mixed together to make a composite sample for further use in the experiment. The soil has been characterized as a calcareous one with very low organic matter content (Table 1).

The biogenic zinc oxide nanoparticles (nZnO) used in this study were primarily synthesized for degradation of dyes present in the wastewater released by textile industry. They showed excellent potential of degrading a multitude of azo dyes^25^. The next logical step was to ascertain its toxicity in soil environment. For synthesis of zinc oxide nanoparticles (nZnO), an NOs synthesizing bacterial strain *Psedochrobactrum* sp. C5 was inoculated in 50 mL nutrient broth medium and incubated for growth under shaking (150 rpm) for 24 h at 28 °C under dark. This 50 mL culture was then added with 0.003 M zinc acetate salt and incubated under shaking at 150 rpm at 28 °C. After 72 h, the culture was collected and cell free supernatant was oven dried at 85 °C. This powder was calcined for 7 h in muffle furnace at 700 °C and then ground into a fine powder. The functional groups present on the nZnO were determined by analyzing the particles in a Fourier Transform Infrared spectroscopy (FTIR- Bruker TENSOR-27) in the spectral range of 2000–500 cm^−1^. Particle morphology and micro structure of the nZnO were determined by field emission scanning electron microscopy (FESEM, LEO 1530, Germany). X-ray diffraction was employed to estimate the crystallinity of the nZnO. The zeta potential of the nZnO was determined by dynamic light scanning technique (Zeta PALS, Brookhaven Instrument Corp., Holtsville, NY, USA) after the nZnO particles were dispersed in distilled water and sonicated for five minutes to break the bonds between the particles.

Dried rice straw was used to make biochar using the process described previously by our lab^17^. Briefly, well dried small pieces of rice straw were placed in a Pyrex flask of 2L inside a muffle furnace. The pyrolysis was performed 550 °C with the heating rate of approximately 10 °C min^−1^.

### Soil incubation

An incubation experiment was carried out where soils amended with zinc oxide nanoparticles (nZnO) in the presence or absence of rice-straw derived biochar (BC) were incubated for 64 days. For each treatment, eighty grams dry weight equivalent of fresh soil were weighed in glass beakers. After adjusting the moisture content of the soils at 60% of the water holding capacity, soils were sealed in 1L Mason jars and pre-incubated at 20 °C for two weeks. The pre-incubation was meant for the soil microbial activity to stabilize^45^. Incubations were in triplicates with three levels of nZnO (0, 100, 1000 mg/kg soil) and BC (0, 1, and 5% on weight basis). These levels of NP addition were chosen based on the range i.e. 100–6400 mg kg^−1^ soil for assessing toxicity to soil processes, as previously described^11,46^. At the end of the pre-incubation, soils were amended with nZnO and/or BC. The nanoparticles and biochar were mixed with soil in powdered form. Un-amended soils, which were used as controls, were also mixed using spatula to apply uniform soil disturbance across all the treatments. All the treated and control soils were incubated along with one vial of 10 ml 1 M NaOH to capture the CO_2_ emissions. Another vial containing 10 ml distilled water was placed inside the incubation jars to avoid drought in the mason jars. Sealed Jars having NaOH and 10 ml distilled water, with no soil containing microcosms, were used as blanks for the correction of ambient CO_2_ concentration. The collected samples were titrated for excess of NaOH against 1 M HCl solution to measure CO_2._ Thereafter, the mason jars were sealed tightly with screw lids to avoid CO_2_ leakage. In order to measure C mineralization (CO_2_ emissions from soils), the NaOH containing vials were routinely taken out for CO_2_ measurement and were replaced with fresh NaOH-containing vials in mason jars. The harvested NaOH was titrated with 1 M HCl after precipitating the carbonates by adding 2 ml of BaCl_2_. Two drops of phenolphthalein were added as indicator to determine the excess non-reacted NaOH, on the basis of which CO_2_-C trapped in NaOH was determined^47,48^. At each NaOH replacement, the soil water content was adjusted to 60% of water holding capacity by adding distilled water whenever it was needed based on the gravimetric measurement.

### Soil analyses

Soil was harvested twice for different analyses. First aliquots of about 25 g fresh soil were taken out from microcosms after 24d of incubation. The second harvest was done at the end of the experiment i.e. after 64d of incubation. Microbial biomass C and dissolved organic C were measured on both harvests whereas all the other soil analyses were done only on final harvest.

Fumigation extraction method was used to determine the microbial biomass C^49^. Ten g of moist soil taken from each microcosm was split into two parts. One part of 5 g moist soil was fumigated with ethanol free CHCl_3_ in a vacuum desiccator for 48 h at 25 °C. After removing the fumigants, 25 mL of 0.5 M K_2_SO_4_ was used to extract each sample after shaking for 30 min on a reciprocal shaker. Whatman filter paper were used to filter the extracts. Similar method was used to extract non-fumigated soil^49^ . The dissolved organic carbon in the extracts was determined by using the modified Walkley–Black method^50,51^. The difference of soluble C between fumigated and non-fumigated samples after accounting for an extraction factor of 0.45 was recorded as MBC^52^. The C concentration from the non-fumigated soil samples was considered as dissolved organic carbon (DOC)^53^. We also calculated the metabolic quotient (qCO_2_) from the method adapted from Dilly and Munch (Dilly and Munch 1998) who defined this as a ratio between respiration rate and microbial biomass carbon. The C mineralization measured after 24d & 64d of incubation were used to calculate the metabolic quotient.

Soil pH was measured using a Jenway pH meter by mixing soil with distilled water (1:5, w: v ratio) and shaking the suspension for 30 min on a shaker. The nitrate content in soil solution (NO_3_-N) were determined using the salicylic acid nitration method^54^. Briefly, 10 g of fresh soil was extracted with 20 ml of 0.5 M K_2_SO_4_ after shaking for 30 min at 60 rpm. The extracts were filtered through N-free filter paper. A 0.5 ml of the filtrate and standard (prepared using KNO_3_) was pipetted in clearly labelled test tubes. One ml of 5% salicylic acid solution prepared in sulfuric acid was added to each test tube followed by mixing on a vortex mixer and a rest of 30 min. Afterwards, 10 ml of 4 M NaOH was added to each test tube followed by a rest of 1 hr for full color development. The absorbance was then read on a UV–visible spectrophotometer at 410 nm.

The ammonium content in soil solution (NH_4_^+^- N) were determined by using Indophenol blue method^55^. Briefly, 5 g of fresh soil were extracted with 20 ml of 2 M KCl after 1 hr of shaking. Calibration standards were prepared with an ammonium salt. For photometric analysis, 5 ml of filtrate (or standard) were pipetted into a test tube. Two and half ml of a reagent that was prepared by mixing equal volumes of 0.3 M NaOH and 1.06 M sodium salicylate was added to the test tubes. Moreover, 1 ml of 39.1 mM sodium dichloroisocyanurate solution was also added. The mixture was shaken well and allowed to stand for 30 min at room temperature. The extinction of samples and the standards was read against the reagent blank at 662 nm on a UV–visible spectrophotometer. Net N mineralization for each treatment was quantified by subtracting extractable mineral N contents of the un- amended control soil from extractable mineral N contents found in each treatment^56^.

Available phosphorus in soil extracts was determined photometrically at 882 nm as a blue phosphate molybdic acid complex^57^. Briefly, 5 g of moist soil samples were extracted with 100 ml of 0.5 M sodium bicarbonate solution after shaking end-over-end for 30 min. A 5 ml of filtrate was pipetted into a beaker followed by adjustment of its pH at 5 with sulfuric acid and making up the volume to 20 ml. Sixteen ml of 0.001 M ammonium heptamolybdate working solution were added to the mix followed by the addition of 2 ml ascorbic acid. All the samples, controls and calibration standards were prepared in the similar way. After 15 min, color complex of the solutions were measured at 882 nm on a UV–Visible spectrophotometer.

Cultivable heterotrophic bacteria were determined using the pour plate method^27^.. One g of fresh soil was suspended in 99 ml buffered peptone water in 250 ml a conical flask. After shaking flasks on an orbital shaker at 140 rpm for 30 min, serial dilutions were prepared and poured on nutrient agar. These inoculated plates were inoculated at 28 °C for 3 days. After incubation, the colony forming units (cfu ml^−1^) were estimated by colony counter.

### Statistical analysis

Multiple factor analysis of variance (ANOVA) was used to assess the effect of nZnO, BC and their interaction (i.e. nZnO × BC) on soil pH, microbial biomass C, dissolved organic C, metabolic quotient, ammonium, nitrate, mineral nitrogen, available phosphorus and bacterial colony forming units. In order to determine the treatment effects on carbon mineralization (rate & cumulative), multiple factor ANOVA with nZnO, BC, time since incubation and their interaction as main factors was used. For both sets of ANOVAs used in the study, Post-hoc HSD test was used to distinguish the significantly different means at 95% confidence interval. All the statistical tests were performed using the STATGRAPHICS Centurion XVI software.

## Supplementary Information

Supplementary Information

## References

[1] Klaine SJ (2008). Nanomaterials in the environment: Behaviour, fate, bioavailability, and effects. Environ. Toxicol. Chem..

[2] Seleiman MF (2021). Nano-fertilization as an emerging fertilization technique: why can modern agriculture benefit from its use?. Plants.

[3] Gruère G, Narrod C, Abbott L (2011). Agricultural, food, and water nanotechnologies for the poor.

[4] Dror I, Yaron B, Berkowitz B (2015). Abiotic soil changes induced by engineered nanomaterials: a critical review. J. Contam. Hydrol..

[5] Kah, M., Beulke, S., Tiede, K. & Hofmann, T. Nanopesticides: state of knowledge, environmental fate, and exposure modeling. *Crit. Rev. Environ. Sci. Technol.* (2013).

[6] Zabrieski Z (2015). Pesticidal activity of metal oxide nanoparticles on plant pathogenic isolates of Pythium. Ecotoxicology.

[7] Hu CW (2010). Toxicological effects of TiO2and ZnO nanoparticles in soil on earthworm Eisenia fetida. Soil Biol. Biochem..

[8] Parada J (2019). The nanotechnology among US: are metal and metal oxides nanoparticles a nano or mega risk for soil microbial communities?. Crit. Rev. Biotechnol..

[9] Seleiman MF (2020). Effects of ZnO nanoparticles and biochar of rice straw and cow manure on characteristics of contaminated soil and sunflower productivity, oil quality, and heavy metals uptake. Agronomy.

[10] Hermes P-H, Fabián F-L, Esperanza H-L, Jorge M-V, David Á-SJ (2020). Effect of engineered nanoparticles on soil biota: do they improve the soil quality and crop production or jeopardize them?. L. Degrad. Dev..

[11] Rashid MI (2016). Zinc oxide nanoparticles affect carbon and nitrogen mineralization of Phoenix dactylifera leaf litter in a sandy soil. J. Hazard. Mater..

[12] Shahzad T (2015). Contribution of exudates, arbuscular mycorrhizal fungi and litter depositions to the rhizosphere priming effect induced by grassland species. Soil Biol. Biochem..

[13] Dumont E, Johnson AC, Keller VDJ, Williams RJ (2015). Nano silver and nano zinc-oxide in surface waters—exposure estimation for Europe at high spatial and temporal resolution. Environ. Pollut..

[14] Prasanna, S. R. V. S., Balaji, K., Pandey, S. & Rana, S. Chapter 4 - Metal Oxide Based Nanomaterials and Their Polymer Nanocomposites. in (ed. Karak, N. B. T.-N. and P. N.) 123–144 (Elsevier, 2019). https://doi.org/10.1016/B978-0-12-814615-6.00004-7

[15] Dinesh R, Anandaraj M, Srinivasan V, Hamza S (2012). Engineered nanoparticles in the soil and their potential implications to microbial activity. Geoderma.

[16] Jeffery S, Verheijen FGA, van der Velde M, Bastos AC (2011). A quantitative review of the effects of biochar application to soils on crop productivity using meta-analysis. Agric. Ecosyst. Environ..

[17] Riaz M (2017). Corncob-derived biochar decelerates mineralization of native and added organic matter (AOM) in organic matter depleted alkaline soil. Geoderma.

[18] Seleiman MF (2019). Integrative effects of rice-straw biochar and silicon on oil and seed quality, yield and physiological traits of *Helianthus annuus* L. grown under water deficit stress. Agronomy.

[19] O’Connor D (2018). Biochar application for the remediation of heavy metal polluted land: a review of in situ field trials. Sci. Total Environ..

[20] Zhang X (2013). Using biochar for remediation of soils contaminated with heavy metals and organic pollutants. Environ. Sci. Pollut. Res..

[21] Gonçalves SPC, Strauss M, Martinez DST (2018). The positive fate of biochar addition to soil in the degradation of PHBV-silver nanoparticle composites. Environ. Sci. Technol..

[22] Taş AC, Majewski PJ, Aldinger F (2002). Synthesis of gallium oxide hydroxide crystals in aqueous solutions with or without urea and their calcination behavior. J. Am. Ceram. Soc..

[23] Huang J (2007). Biosynthesis of silver and gold nanoparticles by novel sundried Cinnamomum camphora leaf. Nanotechnology.

[24] Nagarajan S, Kuppusamy KA (2013). Extracellular synthesis of zinc oxide nanoparticle using seaweeds of gulf of Mannar, India. J. Nanobiotechnol..

[25] Siddique K (2021). Comparative efficacy of biogenic zinc oxide nanoparticles synthesized by Pseudochrobactrum sp. C5 and chemically synthesized zinc oxide nanoparticles for catalytic degradation of dyes and wastewater treatment. Environ. Sci. Pollut. Res..

[26] Ge Y, Schimel JP, Holden PA (2011). Evidence for negative effects of TiO_2_ and ZnO nanoparticles on soil bacterial communities evidence for negative effects of TiO_2_ and ZnO nanoparticles on soil bacterial communities. Environ. Sci. Technol..

[27] Rashid MI (2017). Toxicity of iron oxide nanoparticles to grass litter decomposition in a sandy soil. Sci. Rep..

[28] Jośko I, Oleszczuk P, Futa B (2014). The effect of inorganic nanoparticles (ZnO, Cr2O3, CuO and Ni) and their bulk counterparts on enzyme activities in different soils. Geoderma.

[29] Asadishad B (2018). Amendment of agricultural soil with metal nanoparticles: effects on soil enzyme activity and microbial community composition. Environ. Sci. Technol..

[30] Jośko I (2019). Long-term effect of ZnO and CuO nanoparticles on soil microbial community in different types of soil. Geoderma.

[31] Khan M, Scullion J (2002). Effects of metal (Cd, Cu, Ni, Pb or Zn) enrichment of sewage-sludge on soil micro-organisms and their activities. Appl. Soil Ecol..

[32] Biederman LA, Harpole WS (2013). Biochar and its effects on plant productivity and nutrient cycling: a meta-analysis. GCB Bioenergy.

[33] Dempster DN, Gleeson DB, Solaiman ZM, Jones DL, Murphy DV (2012). Decreased soil microbial biomass and nitrogen mineralisation with Eucalyptus biochar addition to a coarse textured soil. Plant Soil.

[34] Yin Y, He X, Gao R, Ma H, Yang Y (2014). Effects of rice straw and its biochar addition on soil labile carbon and soil organic carbon. J. Integr. Agric..

[35] García-gómez C (2018). Soil pH effects on the toxicity of zinc oxide nanoparticles to soil microbial community. Environ. Sci. Pollut. Res..

[36] Sri Sindhura K, Prasad TNVKV, Panner Selvam P, Hussain OM (2014). Synthesis, characterization and evaluation of effect of phytogenic zinc nanoparticles on soil exo-enzymes. Appl. Nanosci..

[37] Bardgett RD, Saggar S (1994). Effects of heavy metal contamination on the short-term decomposition of labelled [14C]glucose in a pasture soil. Soil Biol. Biochem..

[38] Dornbush ME (2007). Grasses, litter, and their interaction affect microbial biomass and soil enzyme activity. Soil Biol. Biochem..

[39] Jiang X, Haddix ML, Cotrufo MF (2016). Interactions between biochar and soil organic carbon decomposition: effects of nitrogen and low molecular weight carbon compound addition. Soil Biol. Biochem..

[40] McDonald GK (2017). A survey of total and dissolved organic carbon in alkaline soils of southern Australia. Soil Res..

[41] El-Naggar AH (2015). Carbon mineralization and nutrient availability in calcareous sandy soils amended with woody waste biochar. Chemosphere.

[42] Zhu D (2020). Synthesis and characterization of magnesium oxide nanoparticle-containing biochar composites for efficient phosphorus removal from aqueous solution. Chemosphere.

[43] Bruinink A, Wang J, Wick P (2015). Effect of particle agglomeration in nanotoxicology. Arch. Toxicol..

[44] Zare Y, Rhee KY, Hui D (2017). Influences of nanoparticles aggregation/agglomeration on the interfacial/interphase and tensile properties of nanocomposites. Compos. Part B Eng..

[45] Shahzad T (2018). Root penetration in deep soil layers stimulates mineralization of millennia-old organic carbon. Soil Biol. Biochem..

[46] Waalewijn-Kool PL, Diez Ortiz M, van Gestel CAM (2012). Effect of different spiking procedures on the distribution and toxicity of ZnO nanoparticles in soil. Ecotoxicology.

[47] Rehman, K. *et al.* Effect of Reactive Black 5 azo dye on soil processes related to C and N cycling. *PeerJ* **2018**, (2018). 10.7717/peerj.4802 PMC5969049 29844965

[48] Zibilske, L. M. Carbon mineralization. in *Methods of Soil Analysis Part 2, Microbiological and Biochemical Properties* (eds. Weaver, R. W. et al.) 835–864 (SSSA, 1994).

[49] Vance ED, Brookes PC, Jenkinson DS (1987). An extraction method for measuring soil microbial biomass C. Soil Biol. Biochem..

[50] Jackson, M. L. Soil chemical analysis, constable and Co. *Ltd. London* **497**, (1962).

[51] Walkley A, Black IA (1934). An examination of the Degtjareff method for determining soil organic matter, and a proposed modification of the chromic acid titration method. Soil Sci..

[52] Joergensen RG (1996). The fumigation-extraction method to estimate soil microbial biomass: Calibration of the kEC value. Soil Biol. Biochem..

[53] Jones DL, Willett VB (2006). Experimental evaluation of methods to quantify dissolved organic nitrogen (DON) and dissolved organic carbon (DOC) in soil. Soil Biol. Biochem..

[54] Cataldo DA, Maroon M, Schrader LE, Youngs VL (1975). Rapid colorimetric determination of nitrate in plant tissue by nitration of salicylic acid. Commun. Soil Sci. Plant Anal..

[55] Keeney, D. R. & Nelson, D. W. Nitrogen - Inorganic forms. in *Methods of Soil Analysis, Part 2* (eds. Page, A. L. & Miller, R. H.) 643–698 (American Society of Agronomy, 1982).

[56] Sierra J (1997). Temperature and soil moisture dependence of N mineralization in intact soil cores. Soil Biol. Biochem..

[57] Olsen SR, Sommers LE, Page AL (1982). Methods of soil analysis. Part.

