# Supplementary material for: Toxicity of biogenic zinc oxide nanoparticles to soil organic matter cycling and their interaction with rice-straw derived biochar
Source: Sci Rep. 2021 Apr 19;11:8429. doi: 10.1038/s41598-021-88016-x (PMC8055651; doi:10.1038/s41598-021-88016-x)
Supplement: Supplementary file 1 — Supplementary Information [file 41598_2021_88016_MOESM1_ESM.pdf]

Toxicity of biogenic zinc oxide nanoparticles to soil organic matter cycling and their interaction with rice-straw derived biochar

Shemawar[1], Abid Mahmood[1], Sabir Hussain[1], Faisal Mahmood[1], Muhammad Iqbal[1], Muhammad Shahid[2], Muhammad Ibrahim[1], Muhammad Arif Ali[3], Tanvir Shahzad[1*]

[1]Department of Environmental Sciences & Engineering, Government College University Faisalabad, Allama Iqbal Road, 38000 Faisalabad, Pakistan

[2] Department of Bioinformatics & Biotechnology, Government College University Faisalabad, Allama Iqbal Road, 38000 Faisalabad, Pakistan

[3] Department of Soil Science, Bahauddin Zakariya University Multan, Pakistan

** Author for correspondence:*

Tanvir Shahzad,

Email: hereistanvir@gmail.com, tanvirshahzad@gcuf.edu.pk, Phone: +92 308 4508683

Type of article: Original full length paper

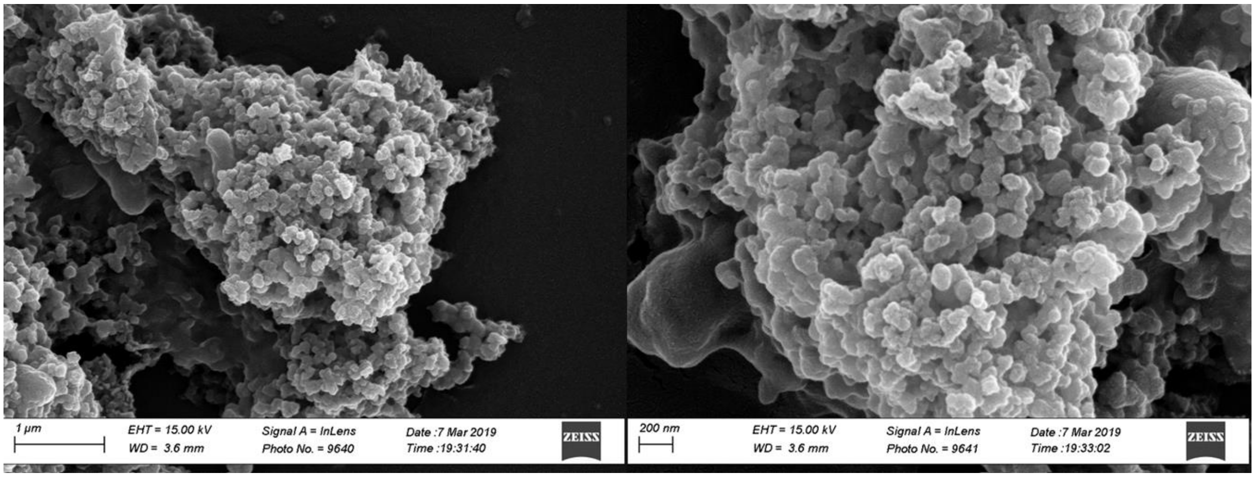

Fig S1. FESEM images of biosynthesized nZnO at different magnifications (First published elsewhere[1])

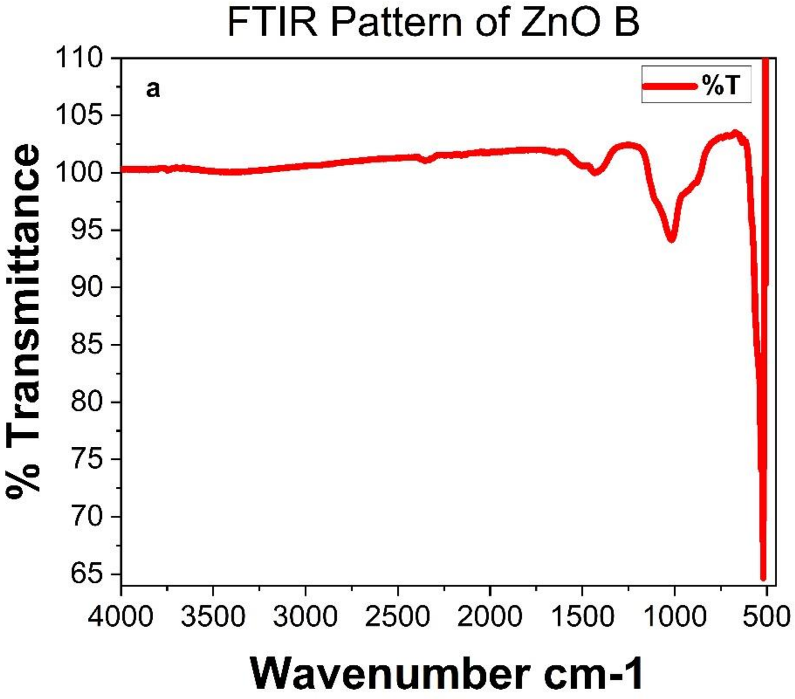

Fig. S2. FTIR spectra of biosynthesized synthesized nZnO[1]

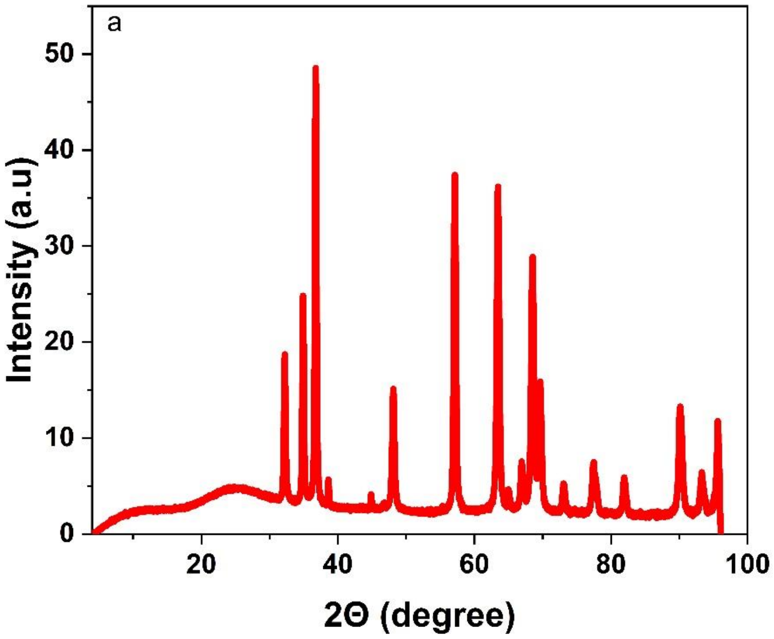

Fig. S3. XRD spectra of biosynthesized synthesized nZnO[1]

Reference Cited:

1. Siddique, K. *et al.* Comparative efficacy of biogenic zinc oxide nanoparticles synthesized by Pseudochrobactrum sp. C5 and chemically synthesized zinc oxide nanoparticles for catalytic degradation of dyes and wastewater treatment. *Environ. Sci. Pollut. Res.* (2021). doi:10.1007/s11356-021-12575-9
